# Clinical pathogen profiles and lung microbiome features in lung infection patients and concurrent cancer: insights from metagenomics next-generation sequencing

**DOI:** 10.1515/biol-2025-1220

**Published:** 2026-03-04

**Authors:** Dandan Liu, Yuehong Ma, Qian Ma, Huihua Huang, Tianyu Li, Jin Wang, Jin Zhang, Xuesong Cheng, Xueru Ge, Yan Chen, Yanbei Zhang

**Affiliations:** Department of Pulmonary and Critical Care Medicine, Anhui No.2 Provincial People’s Hospital, Hefei, China; Department of Pulmonary and Critical Care Medicine, The First Affiliated Hospital of Anhui Medical University, 218 Jixi Road, Shushan District, Hefei, China; Anhui Province Key Laboratory of Occupation Health, Anhui No.2 Provincial People’s Hospital, Hefei, China; Genoxor Medical Science and Technology Inc., Shanghai, China; Department of Pharmacy, Shanghai Sixth People’s Hospital Affiliated to Shanghai Jiao Tong University School of Medicine, Shanghai, China

**Keywords:** pulmonary infection, pathogen spectrum, mNGS, cancer patient, non-albicans Candida

## Abstract

Pulmonary infections in immunocompromised cancer patients present significant diagnostic and therapeutic challenges. From Dec 2021 to Aug 2023, 85 patients with pulmonary infection were enrolled and categorized into a cancer group (CP, *n* = 20) and a non-cancer control group (NCP, *n* = 18). Pathogen detection was performed using both mNGS and culture and lung microbiome analysis was conducted. mNGS demonstrated a significantly higher pathogen detection rate than culture (P < 0.0001). The CP group exhibited older age (P < 0.001), elevated neutrophil counts (NE) and higher procalcitonin (PCT) levels compared to the NCP group. Furthermore, fungal pathogens were significantly more prevalent in the CP group (P = 0.046). Both cancer status and advanced age were independent influencing factors for the detection of pulmonary fungi. Pulmonary microbiome analysis revealed no significant differences in *α*-diversity or *β*-diversity between groups. These findings indicate that mNGS offers superior sensitivity over culture. Cancer-related pulmonary infections present a distinct pathogen profile characterized by a higher prevalence of fungal pathogens. This underscores the need for enhanced clinical vigilance, especially among elderly cancer patients.

## 1 Introduction

Pulmonary infections represent a major cause of global mortality and morbidity, particularly among immunocompromised individuals [1]. Such patients are at high risk for various types of infections, including opportunistic and polymicrobial infections. [2]. Cancer patients are especially prone to pulmonary infections due to factors such as advanced age, immunosuppression, chemotherapy, and malnutrition. Multiple studies have indicated that cancer patients are more susceptible to COVID - 19 than the general population, and solid cancer have been identified as an independent predictor of poor prognosis and in–hospital mortality in COVID - 19 patients [3], [4], [5]. Dandachi et al. [6] further recommended that solid tumor patients with underlying pulmonary diseases should be vigilant against invasive pulmonary aspergillosis. Pulmonary infection also constitute a leading cause of death in patients with hematological malignancies, particularly those who have undergone high–dose chemotherapy or hematopoietic stem cell transplantation (HSCT) [7], 8]. Although current research has improved our understanding of risk factors and common pathogens associated with pulmonary infections in cancer patients, challenges remain in diagnosis due to atypical clinical presentations and complex patient backgrounds. Therefore, rapid and accurate identification of causative pathogens is essential for effective management of pulmonary infections and for improving patient outcomes [9]. However, conventional diagnostic methods, which rely heavily on culture-based techniques, are limited by their sensitivity, turnaround time, and spectrum of detectable pathogen. These limitations pose a major obstacle to achieving timely and precise diagnosis of infectious diseases [10], 11].

Metagenomic next-generation sequencing (mNGS) has emerged as a powerful tool for detecting infectious agents, including those that are rare or emerging, such as Severe Acute Respiratory Syndrome Coronavirus 2, *Legionella pneumophila*, and *Cryptococcus neoformans* [12], [13], [14]. By enabling high-throughput sequencing of millions to billions of DNA fragments, mNGS allows comprehensive and unbiased detection of pathogens through computational alignment reference genomic databases [15]. Empirical studies have demonstrated the utility of mNGS in diagnosing infections in diverse clinical settings – including the central nervous system, lungs, bloodstream, and eyes [16], [17], [18], [19]. However, there is limited literature on the application of mNGS for detecting pathogens in cancer patients with pulmonary infections.

Beyond pathogen identification, mNGS also provides a means to characterize the local microbiome at the infection site. The respiratory tract constitutes a polymicrobial ecological niche, where disruptions in microbial composition are frequently associated with respiratory diseases. One study suggests that the pathogenesis of pneumonia may involve a rapid transition from a stable pulmonary microbiota to a dysbiotic state, marked by reduced microbial diversity, increased microbial burden, and heightened host inflammation [20]. Nevertheless, the relationship between respiratory microbiome dynamics and pulmonary infections in cancer patients remains poorly understood.

In light of these challenges, this study used mNGS to conduct a comprehensively analysis of respiratory samples from cancer patients with pulmonary infections. Our objectives were to establish an accurate profile of potential pathogens, characterize the structure of the lung microbiota, and integrate clinical metadata to support more more targeted preventive and treatment strategies.

## 2 Methods

### 2.1 Study design and samples collection

**Figure 1:**
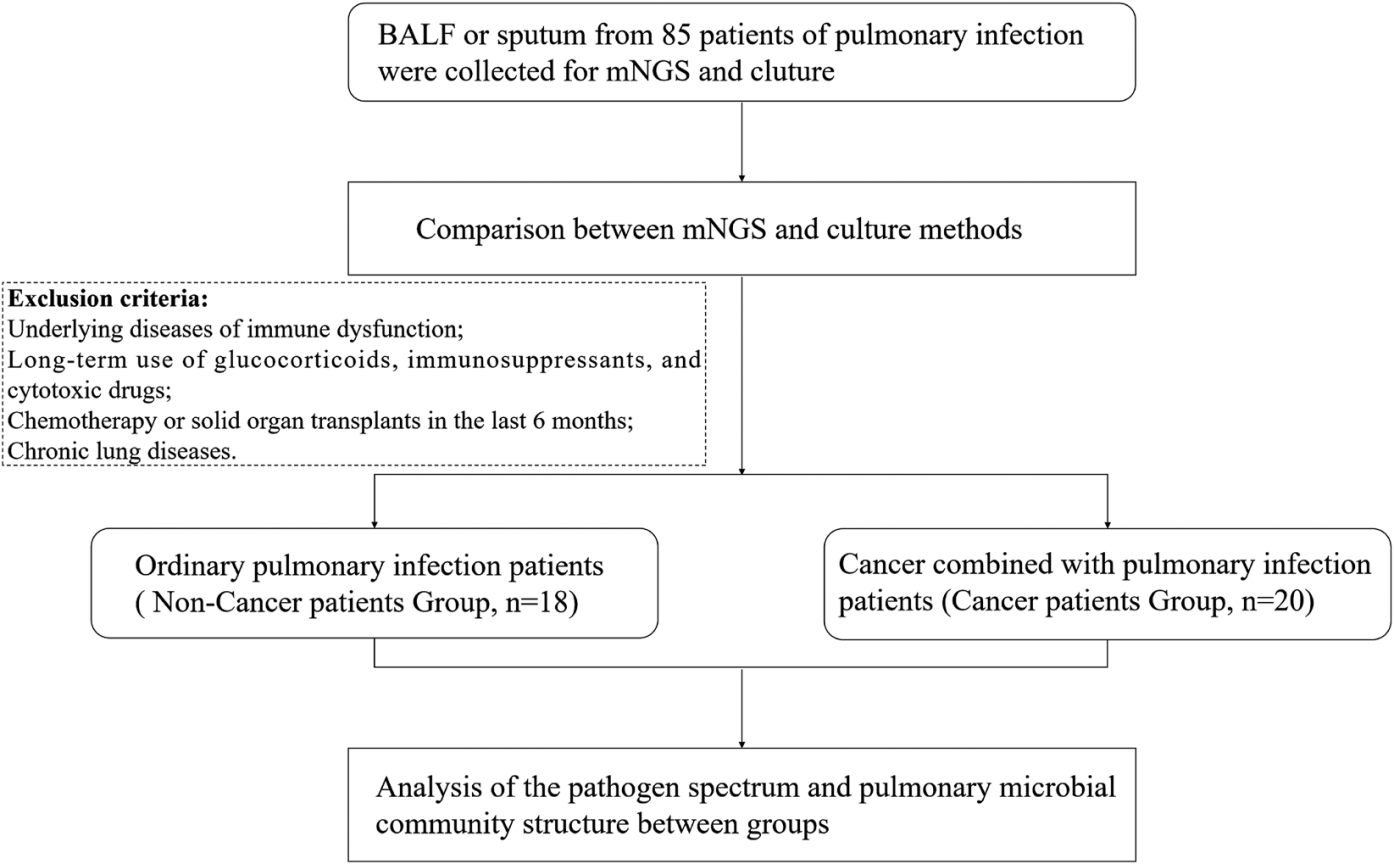
Overview of patient enrollment workflow.

Between December 2021 and August 2023, Patients diagnosed with pulmonary infections were recruited from the Department of Pulmonary and Critical Care Medicine at Anhui No.2 Provincial People’s Hospital. This study was conducted in accordance with the Declaration of Helsinki and received approval from the Hospital’s Ethics Committee. A total of 85 patients with lung infections were initially enrolled (Figure 1). The inclusion criteria were as follows: (i) clinical diagnosis of pulmonary infection; (ii) consent for the collection of respiratory samples for pathogen detection via mNGS and culture tests; and (iii) availability of complete clinical records. Patients who did not meet all these criteria were excluded.

Participants were divided into two groups based on underlying diseases and immune status: those with ordinary pulmonary infections (non-cancer patients, NCP) and those with both cancer and pulmonary infection (cancer patients, CP). The NCP group consisted of patients with pulmonary infections but no immunosuppression, meeting the following criteria: (i) no history of immunosuppressive conditions; (ii) no chronic use of immunosuppressive drugs, such as glucocorticoids or steroids; (iii) no chemotherapy or solid organ transplantation within the preceding six months; and (iv) no chronic lung diseases.

**Informed consent:** Informed consent has been obtained from all individuals included in this study.

**Ethical approval:** The research related to human use has been complied with all the relevant national regulations, institutional policies and in accordance with the tenets of the Helsinki Declaration, and has been approved by the Ethics Committee of Anhui No.2 Provincial People’s Hospital (No. 2024-01).

### 2.2 Metagenomic sequencing and bioinformatics analysis

Total DNA was extracted from bronchoalveolar lavage fluid (BALF) and sputum samples using the Tiangen Magnetic DNA Kit (Tiangen, China). DNA libraries were constructed through a multistep process including DNA fragmentation, end-repair, A-tailing add, adapter ligation, and PCR amplification with the NEB Next^®^ Ultra™ DNA Library Prep Kit for Illumina^®^ sequencing. libraries quality was assessed using an Agilent 2100 Bioanalyzer, and concentration was quantified with a Qubit 2.0 Fluorometer. Qualified libraries were subsequently sequenced on the Illumina Next-seq platform.

The bcl2fastq2 v2.20 software was used to split raw sequencing data. Trimmomatic v0.36 software [21] was engaged to trim adaptor sequences and low-quality bases. Human-derived sequences were removed by alignment to the human reference genome using Bowtie v2.2.629 software [22], retaining non-human reads for subsequent microbial analysis. These reads were aligned against a custom microbial genome database comprising over 27,000 species, curated from public reference sources. Microbiota analysis was conducted using the online system “Statistical Analysis and Visualization of Metagenomic Sequencing (SAV-mNGS)” (http://192.168.1.229:3838/SAV-mNGS/), an R Shiny application developed and maintained by Genoxor Medical Science and Technology Inc. (Shanghai, China). Alpha diversity was assessed using the Shannon and Chao1 indices to evaluate microbial diversity and richness, respectively. Beta diversity was examined via Principal Coordinate Analysis (PCoA) and Analysis of Similarities (ANOSIM) based on Bray-Curtis dissimilarity. Additionally, Linear discriminant-analysis Effect-Size (LEfSe) was used to identify differentially abundant taxa between groups.

### 2.3 Criteria for a positive mNGS detection

A positive mNGS result was defined based on thresholds values applied to the number of strictly mapped reads for each type of microorganism. For bacteria, mycoplasma, chlamydia, DNA viruses or fungi, a minimum of three reads was required. For members of the *Mycobacterium tuberculosis* complex (MTC), a minimum of one read was used as the detection threshold.

### 2.4 Microbial culture

To complement the potential pathogen profile and evaluate the positive detection rate of mNGS, results from traditional culture methods-considered the clinical “gold standard” for pathogen identification-were also obtained and analyzed [23], [24], [25].

All bacterial and fungal culture were processed by the Diagnostic Laboratory of Anhui No.2 Provincial People’s Hospital using standard isolation media, including blood agar, Mackcoys agar, chocolate agar, and Mueller-Hinton agar. Cultures were incubated at 37 °C in 5–10 % CO_2_ for 24–48 h using Heal Force HF90 incubator (Heal Force Bio-Meditech Co., Ltd., China). Isolated strains were identified to species level using the VITEK-2 Compact system (bioMérieux, France).

### 2.5 Statistical analyses

Continuous variables are presented as mean ± standard deviation (SD), and categorical variables as counts and percentages. Group comparisons for categorical variables were performed using the Chi-square test or Fisher’s exact test, as appropriate. For continuous variables, the Student’s *t*-test or Mann-Whitney *U* test was applied depending on data distribution. All statistical analyses were performed using GraphPad Prism 7 (GraphPad Prism Software Inc., San Diego, CA). A P value < 0.05 was considered statistically significant.

## 3 Results

### 3.1 Baseline characteristics of all pulmonary infection patients

Of the 85 patients included, 55 males and 30 females, with a median age of 58 years. Hypertension, cancer and diabetes were the most frequently observed comorbidities. Chest radiographs showed bilateral abnormalities in 65 patients (76.47 %) and unilateral abnormalities in 20 patients (23.53 %). The baseline characteristics of the participants are summarized in Table 1.

**Table 1:** Baseline and clinical characteristics of all enrolled patients.

Clinical features	Values
**Characteristics**	
Male, *n* (%)	55 (64.71 %)
Female, *n* (%)	30 (35.29 %)
Age, years (median, range)	58, 14–91
**Underlying diseases, *n* (%)**	
Bronchiectasis	2 (2.35 %)
Bronchial asthma	1 (1.18 %)
COPD	2 (2.35 %)
Respiratory failure	5 (5.89 %)
Cancer	20 (23.53 %)
Pulmonary malignancy	11 (12.94 %)
Hypertension	23 (27.06 %)
Diabetes mellitus	13 (15.29 %)
Cerebrovascular disease	10 (11.76 %)
Chronic gastritis	4 (4.71 %)
Rheumatoid arthritis	2 (2.35 %)
**Abnormality on chest radiograph, *n* (%)**	
Bilateral	65 (76.47 %)
Unilateral	20 (23.53 %)
**Laboratory tests, mean** ± **SD**	
WBC (×109/L)	7.54 ± 4.54
NE (×109/L)	6.65 ± 4.32
CRP (mg/L)	80.58 ± 63.48
PCT (ng/mL)	0.53 ± 7.33

COPD, chronic obstructive pulmonary disease; WBC, white blood cell; NE, neutrophil; CRP, C-reactive protein; PCT, procalcitonin.

### 3.2 Comparison of pathogen detection between mNGS and culture methods

**Figure 2:**
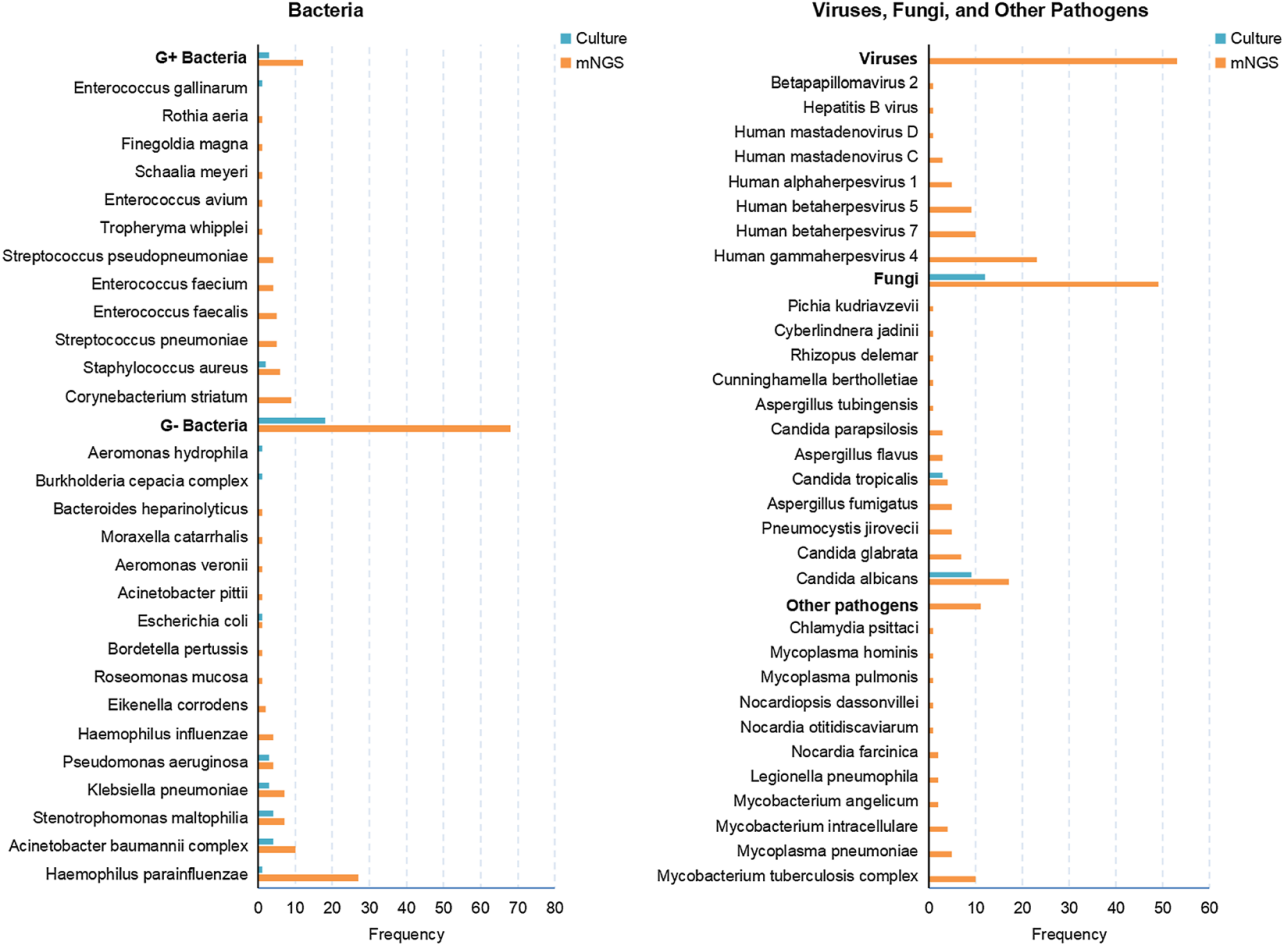
The pathogen spectrum and positive rates of all patients constructed by mNGS and culture tests. G+ Bacteria, gram-positive bacteria; G- bacteria, gram-negative bacteria.

**Figure 3:**
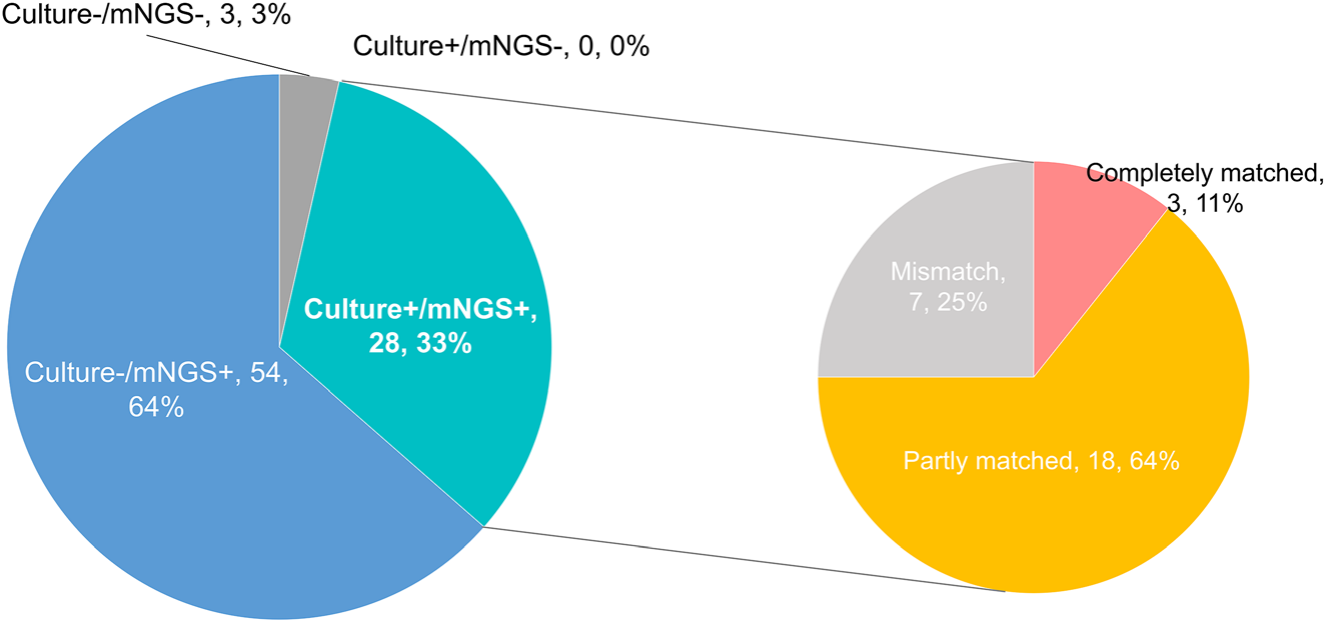
The positivity distribution for detection of pathogen by mNGS and culture. The double-positive samples were further categorized as completely matched, partly matched (at least one overlap of pathogens was observed) and mismatched.

A total of 59 pathogens were identified across all patients. Among these, 56 were detected via mNGS test, whereas only 3 were identified by culture (Figure 2). The most frequently detected bacteria by mNGS were Gram-negative bacteria, including *Haemophilus parainfluenzae*, *Acinetobacter baumannii*, and *Stenotrophomonas maltophilia*. The most common fungi and viruses detected by mNGS were *Candida albicans* and Human gammaherpesvirus 4. respectively. Additionally, mNGS detected fastidious pathogens such as *Mycoplasma*, *Chlamydia*, and *M. tuberculosis*. A significant difference was observed in the pathogen detection rates between mNGS and culture tests (82/85, 96.47 % vs. 28/85, 32.94 %; P < 0.0001). Among the 28 cases positive by culture, complete agreement between mNGS and culture tests occurred in only 3 cases, with 7 cases of disagreement (Figure 3). The remaining 18 cases showed partial concordance, with at least one pathogen common to both mNGS and culture. Three cases tested negative by both methods.

### 3.3 Comparison of clinical characteristics between CP and NCP groups

The baseline demographic and clinical characteristics of the patients in the CP group (*n* = 20) and NCP group (*n* = 18) are summarized in Table 2. The two groups with no significant difference observed in gender distribution (p = 1.000). However, patients in the CP group were significantly older than those in the NCP group (p < 0.001).As expected by the study design, the prevalence of underlying comorbidities differed markedly between the groups. All patients in the CP group had a concurrent cancer diagnosis, whereas none were present in the NCP group (p < 0.001). The most frequent malignancies in the CP group were pulmonary (55 %), followed by gastrointestinal (25 %) and hematological (20 %) malignancies. Consequently, comorbidities such as hypertension (p = 0.001) and kidney disease (p = 0.034) were also significantly more prevalent in the CP group. Radiological presentation was similar between the groups. Laboratory investigations revealed a more pronounced inflammatory response in the CP group. neutrophil (NE) count and procalcitonin (PCT) levels were substantially higher in the CP group (p = 0.026 and 0.016, respectively). Finally, no significant differences were found in the total hospital stay (p = 0.164) or clinical outcomes. A higher proportion of patients in the CP group experienced a worsening clinical course (20 % vs. 5.56 %), but this difference was not statistically significant (p = 0.181). The majority of patients in both groups showed improvement by the end of hospitalization.

**Table 2:** Baseline and clinical characteristics of CP and NCP group.

Clinical features	CP group (*n* = 20)	NCP group (*n* = 18)	*p-*Value
**Characteristics**			
Male, *n* (%)	11 (55 %)	9 (50 %)	1
Age, years (median, range)	65, 37–91	42, 14–76	**<0.001**
**Underlying diseases, *n* (%)**			
Cancer	20 (100 %)	0	<0.001
Pulmonary malignancy	11 (55 %)	0	<0.001
Gastrointestinal malignancy	5 (25 %)	0	0.011
Hematological malignancy	4 (20 %)	0	0.034
Respiratory failure	2 (10 %)	0	0.245
Hypertension	11 (55 %)	0	0.001
Diabetes mellitus	2 (10 %)	0	0.245
Cerebrovascular disease	2 (10 %)	0	0.245
Kidney disease	4 (20 %)	0	0.034
Rheumatoid arthritis	2 (10 %)	0	0.245
**Abnormality on chest radiograph, *n* (%)**			
Bilateral	17 (85 %)	13 (65 %)	0.257
Unilateral	3 (15 %)	7 (35 %)	
**Laboratory tests, mean ± SD**			
WBC (×109/L)	9.20 ± 6.56	5.89 ± 1.88	0.06
NE (×109/L)	7.72 ± 5.87	4.06 ± 1.74	0.026
CRP (mg/L)	53.94 ± 54.25	26.33 ± 29.51	0.075
PCT (ng/mL)	1.00 ± 1.34	0.03 ± 0.04	0.016
**Total hospital stay, mean ± SD**			
Days	14.05 ± 6.82	11.44 ± 3.93	0.164
**Clinical outcome, *n* (%)**			
Worse	4 (20 %)	1 (5.56 %)	0.181
Improved	16 (80 %)	17 (94.44 %)	

WBC, white blood cell; NE, neutrophil; CRP, C-reactive protein; PCT, procalcitonin. The bold values indicate statistical significance.

### 3.4 The potential pathogen spectrum differs between CP and NCP Patients, with a notable lncrease in fungal detection in CP

**Figure 4:**
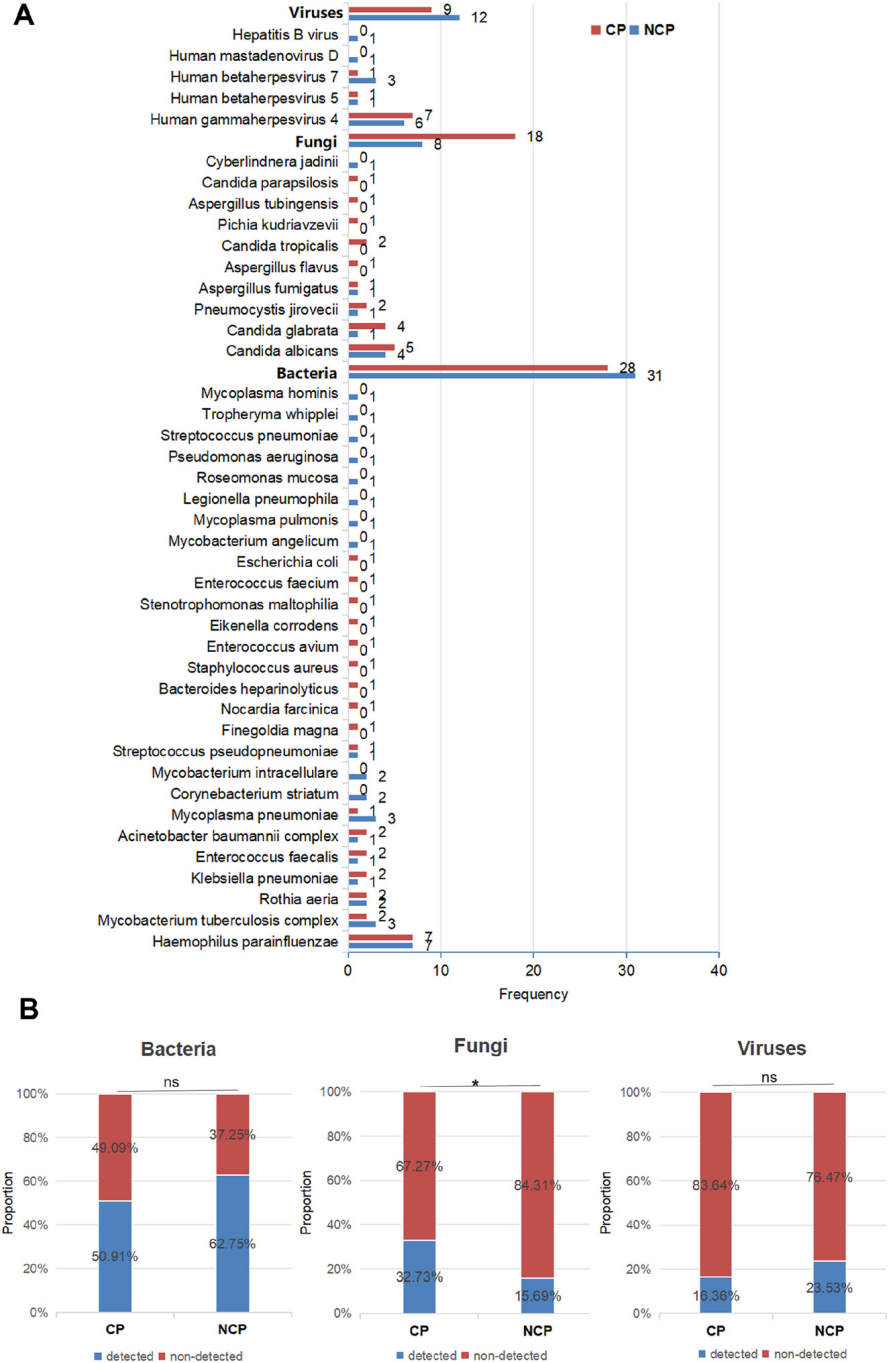
Comparison of pathogen spectrum and detection results obtained by mNGS between the CP and NCP group. (A) The pathogen spectrum of the CP and NCP group constructed. (B) The proportions of bacteria, fungi, and viruses in the total frequency of the CP and NCP groups. Chi–square test was used for statistical analysis. *denotes *p*-Value < 0.05. ns indicates a *p*-Value > 0.05, with no significant difference.

A total of 42 pathogenic species were detected via mNGS in the CP and NCP groups. In the CP group, bacteria, fungi, and viruses were detected 28, 18, and 9 times, respectively. Conversely, in the NCP group, bacteria, fungi, and viruses were detected 31, 8, and 12 times, respectively (Figure 4A). Further analysis revealed differences in the detection frequency of different types of pathogens between the two groups. It is worth noting that a significant difference was observed in the proportion of fungal pathogens within the overall pathogen frequency, with a higher proportion in the CP group (18/55, 32.7 % vs. 9/55, 16.4 %; P = 0.046), while no significant differences were found in the proportions of bacteria and viruses (Figure 4B). Further analysis of fungal pathogens revealed a difference in the detection frequency of non-albicans Candida (NAC) species between the CP and NCP group. NAC was detected 7 times in the CP group (including 4 *Candida glabrata*, 2 *Candida tropicalis*, and 1 *Candida parapsilosis*), while only once in the NCP group (1 *C. parapsilosis*). However, the difference was not reach statistical significant (7/18, 45 % vs. 1/8, 12.5 %; P = 0.179).

We conducted logistic regression analysis to determine the factors affecting positive fungal detection, with cancer, age, lung malignancy and hypertension as variables. The results are presented in Supplementary Table 1. The analysis identified cancer and age as significant predictors of positive fungal detection. Patients with cancer had markedly higher odds (OR = 10.147, p = 0.002). Each additional year of age was associated with a slight increase in odds (OR = 1.050, p = 0.026). Neither lung malignancy (p = 0.271) nor hypertension (p = 0.514) showed a statistically significant association.

### 3.5 The pulmonary microbial community structure of with pulmonary infection and cancer

**Figure 5:**
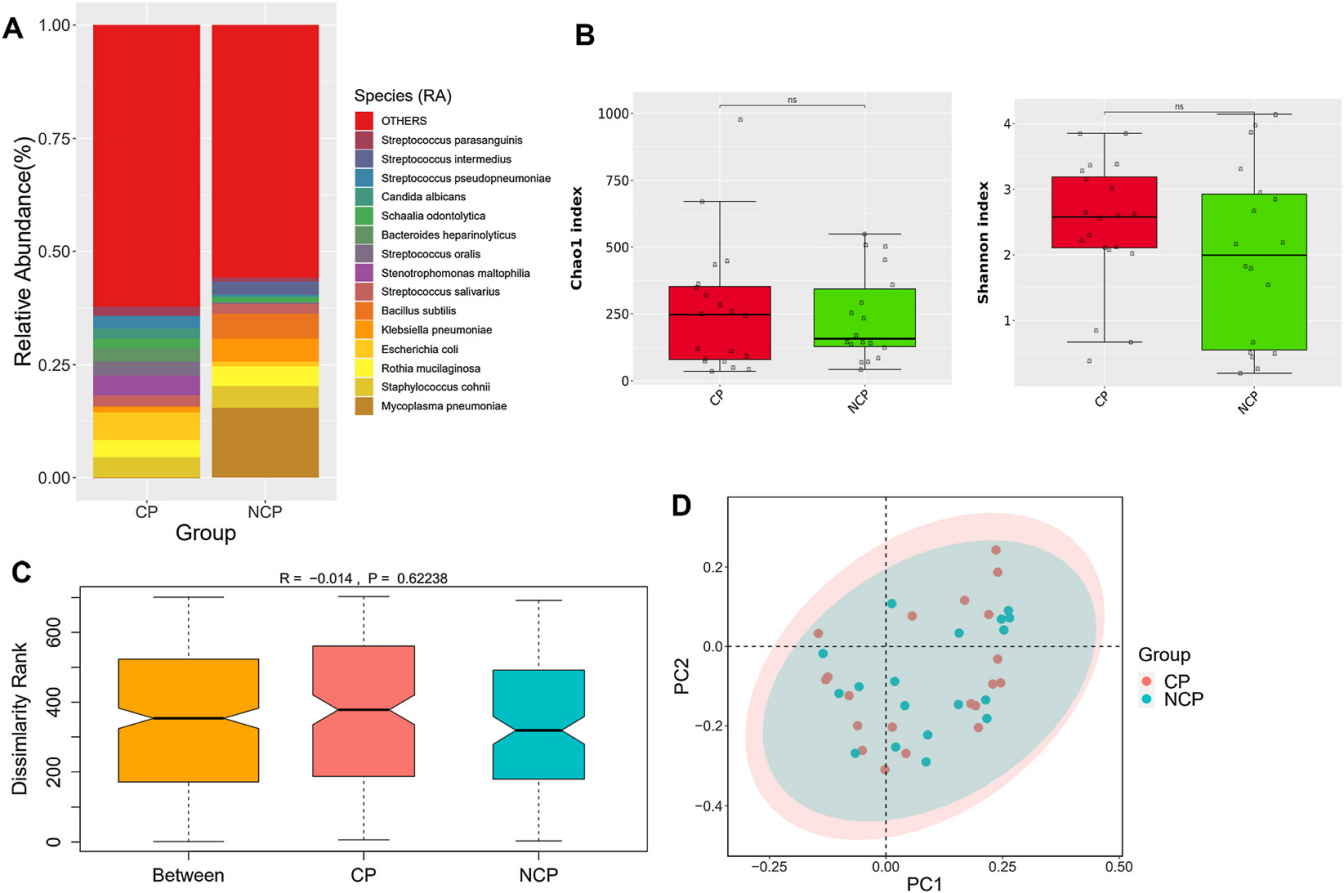
Comparison of respiratory microbiota between CP and NCP groups. (A) Top 15 abundant species in the both groups. (B) Chao1 index and Shannon index. Mann–Whitney *U* test was used for statistical analysis. ns indicates a *p*-Value > 0.05, with no significant difference. (C) ANOSIM boxplot. Beta diversity was assessed by Bray–Curtis measure. (D) PCoA plot.

We used metagenomic data to characteristize the pulmonary microbial communities, with a specific focus on their role in patients with immunosuppressive lung infections associated with cancer. Figure 5A depicts the species with the highly relative abundance in both the CP and NCP groups, which primarily included *Mycoplasma pneumoniae*, *Staphylococcus cohnii*, and *Escherichia coli*. The abundances of *Mycoplasma pneumoniae*, *Klebsiella pneumoniae*, and *Bacillus subtilis* were significantly higher in the NCP group than in the CP group, whereas *E. coli* and *S. maltophilia* were abundant in the CP group. Although *α*-diversity indices (Chao1 and Shannon) suggested greater microbial richness and diversity in the CP group compared to the NCP group, these differences were not statistically significant (P = 0.851 and P = 0.276, respectively; Figure 5B). ANOSIM and PCoA analyses further revealed no significant differences in the microbial community structures between the groups, with no obvious grouping and clustering trend between groups (Figure 5C and D).

## 4 Discussion

Although microbial culture has limitations in detecting viruses, fungi, and fastidious bacteria, it remains a cornerstone of clinical diagnostics by providing live isolates for antimicrobial susceptibility testing. In this study, culture results served as a reference to contextualize the findings from the mNGS assay. Our findings demonstrate a striking disparity in pathogen detection capability between mNGS and conventional culture methods. The significantly higher detection rate of mNGS (96.47 % vs. 32.94 %, p < 0.0001) underscores its superior sensitivity, particularly for pathogens that are fastidious, slow-growing, or non-culturable. This is consistent with previous studies highlighting the utility of mNGS in detecting diverse pathogens directly from clinical samples [26], [27], [28], [29]. The broad spectrum of pathogens identified by mNGS-including fungi, viruses, and intracellular bacteria – illustrates its comprehensive diagnostic capacity. This is especially valuable in immunosuppressed patients, such as those with cancer, where opportunistic infections are common and timely etiological diagnosis is critical [30], 31].

Our study confirms that cancer patients with pulmonary infections exhibit a distinct pathogen profile. A key finding was the increased prevalence of fungal pathogens, particularly non-albicans Candida, in the CP group. This observation is consistent with the report by Zhan et al. [32], who found a higher infection rate of *Pneumocystis jirovecii* in immunocompromised (ICO) patients with pneumonia. Fungi are opportunistic pathogens that commonly colonize human skin and mucous membranes. Under conditions of impaired immunity, they can invade the host and cause opportunistic infections. Consequently, cancer patients are at significantly elevated risk of fungal infections. A previous study reported that 11.6 % of patients with malignant tumors develop hospital-acquired fungal infections, with the respiratory tract being the primary site of infection [33]. Furthermore, compared to patients with other primary tumor sites, lung cancer patients are particularly susceptible to pulmonary fungal infections due to concurrent disruptions and respiratory system disorders [34], [35], [36]. This highlights that cancer patients are a high-risk group for fungal infections.

An important consideration is the significant baseline difference in age between our cohorts. Multivariable regression analysis revealed that both advanced age and cancer status are independent risk factors for fungal detection. This is highly clinically relevant, as older age is a common feature of many cancer populations [37], 38]. The convergence of immunosenescence (age-related decline in immune function) and cancer-associated immunosuppression likely creates a perfect storm, significantly elevating the risk of opportunistic fungal infections in this demographic [39]. Therefore, our results suggest that clinicians should be particularly vigilant about fungal pathogens, especially in the growing population of older oncology patients.

Numerous studies on cancer patients have also identified *Candida* as a common fungal pathogen [34], 40], 41]. *C. albicans* infection can induce acute lung injury in immunosuppressed individuals [42]. Thus, in cancer patients with impaired immune surveillance and defense functions, *Candida* is more likely to invade the respiratory tract, colonize in the lungs, and provoke infection–though the detailed pathogenesis remains unclear. Further analysis showed a higher frequency of NAC species (mainly *C. glabrata* and *C. tropicalis*) in the CP group, although this difference did not reach a statistically significance. Another study reported that NAC species are the predominant cause of invasive Candida infections in intensive care units, possibly due to the widespread use of azole antifungal drugs [43]. Notably, *Candida glabricans* has been identified as the most common NAC among patients with solid tumors and hematological malignancies [44], 45]. This underscores the necessity for accurate identification, tailored antifungal management, and novel treatment strategies against increasingly prevalent drug-resistant NAC species, particularly *C. glabrata*. These observations collectively indicate that the pathogen profile of pulmonary infections in cancer patients is distinct and warrants specific attention in clinical practice.

An increasing number of clinical evidence suggests that dysregulation of the pulmonary microbiome is closely associated with cancer and pulmonary infections [46], [47], [48]. Chen et al. [49] reported that patients with malignant pulmonary tumors exhibited reduced microbial diversity in BALF samples compared to those with benign pulmonary diseases. In contrast, our study found that the diversity and richness of the pulmonary microbiome in CP group were slightly higher than those in NCP group, implying that tumor progression may reshape the pulmonary microenvironment in a way that promotes the proliferation of specific microorganisms. Additionally, *β*-diversity analysis showed no significant separation of microbial communities at the species level between the NP group and NCP group. A plausible explanation is that both groups in this study consisted of cancer patients with concurrent pulmonary infection, sharing similar disease backgrounds that may lead to convergent microbial community structures.

Our study has several limitations. First, the groups were not matched for age or all comorbidities, and despite our statistical adjustments for these imbalances, residual confounding cannot be entirely ruled out. However, our regression analysis strengthens the case for an independent effect of cancer status. Second, the sample size, though sufficient to detect major differences, may be underpowered for more subtle associations. Future prospective studies with larger, age-stratified cohorts are warranted to further validate the interplay between age, cancer, and the lung microbiome. Finally, due to limited patient recruitment, we did not perform correlation analyses between primary tumor site (tumor type) and pathogen detection, particularly with respect to fungi. Such investigations should be undertaken in future large-scale studies.

## 5 Conclusions

Our study demonstrates that mNGS offers significantly higher diagnostic sensitivity than conventional culture, particularly for detecting fungal and fastidious pathogens in cancer-associated pneumonia. We identified a distinct pathogen profile in these immunocompromised hosts, characterized by a marked increase in fungal prevalence, especially non-albicans Candida. Both cancer status and advanced age were independent risk factors for pulmonary fungal detection. These findings underscore the clinical value of mNGS in diagnosing opportunistic infections and highlight the need for heightened vigilance and tailored management of fungal pathogens in older cancer patients. Further large-scale studies are warranted to validate these associations and explore targeted treatment strategies.

## Supplementary Material

Supplementary Material
