# Supplementary material for: Clinical pathogen profiles and lung microbiome features in lung infection patients and concurrent cancer: insights from metagenomics next-generation sequencing
Source: Open Life Sci. 2026 Mar 4;21(1):20251220. doi: 10.1515/biol-2025-1220 (PMC12955702; doi:10.1515/biol-2025-1220)
Supplement: Supplementary file 1 — Supplementary Material [file j_biol-2025-1220_suppl_001.docx]

Supplementary Table 1. Results of multivariate logistic regression analysis of factors associated with positive fungal detection (n=38).

| **Variable** | ***Coefficient*** | ***SE*** | ***Wald X^2^*** | ***P value*** | **OR (95% CI)** |
| --- | --- | --- | --- | --- | --- |
| Intercept | -1.862 | 0.983 | 3.587 | 0.058 | 0.155 (0.023-1.063) |
| Cancer | 2.317 | 0.732 | 10.018 | **0.002** | **10.147 (2.416-42.634)** |
| Age | 0.049 | 0.022 | 4.968 | **0.026** | **1.050 (1.006-1.096)** |
| Pulmonary malignancy | 0.791 | 0.719 | 1.210 | 0.271 | 2.205 (0.539-9.023) |
| Hypertension | 0.439 | 0.673 | 0.426 | 0.514 | 1.551 (0.414-5.807) |

SE, standard error; Wald X^2^, Wald Chi-Square; OR, Odds Ratio; 95% CI, 95% Confidence Interval. The bold values indicate statistical significance.
